# Amelioration of azoxymethane induced-carcinogenesis by reducing oxidative stress in rat colon by natural extracts

**DOI:** 10.1186/1472-6882-14-60

**Published:** 2014-02-18

**Authors:** Mostafa I Waly, Amani S Al-Rawahi, Marwa Al Riyami, Mohamed A Al-Kindi, Halima K Al-Issaei, Sardar A Farooq, Ahmed Al-Alawi, Mohammad S Rahman

**Affiliations:** 1 Department of Food Science and Nutrition, College of Agricultural and Marine Sciences, Sultan Qaboos University, P. O. Box 34–123, Al-Khod, Muscat, Oman; 2 Pathology Department, College of Medicine and Health Sciences, Sultan Qaboos University, P. O. Box 34–123, Al-Khod, Muscat, Oman; 3 Department of Biology, College of Science, Sultan Qaboos University, P. O. Box 34–123, Al-Khod, Muscat, Oman

**Keywords:** Azoxymethane, Oxidative stress, Colon cancer

## Abstract

**Background:**

Azoxymethane (AOM) is a potent carcinogenic agent commonly used to induce colon cancer in rats; the cytotoxicity of AOM is considered to mediate oxidative stress. This study investigated the chemopreventive effect of three natural extracts [pomegranate peel extract (PomPE), papaya peel extract (PapPE) and seaweed extract (SE)] against AOM-induced oxidative stress and carcinogenesis in rat colon.

**Methods:**

Eighty Sprague–Dawley rats (aged 4 weeks) were randomly divided into 8 groups (10 rats/group). Control group was fed a basal diet; AOM-treated group was fed a basal diet and received AOM intraperitonial injections for two weeks at a dose of 15 mg/kg bodyweight, whereas the other six groups were received oral supplementation of PomPE, PapPE or SE, in the presence or absence of AOM injection. All animals were continuously fed *ad-libitum* until aged 16 weeks, then all rats were sacrificed and the colon tissues were examined microscopically for pathological changes and aberrant crypt foci (ACF) development, genotoxicity (induced micronuclei (MN) cells enumeration), and glutathione and lipid peroxidation.

**Results:**

Our results showed that AOM-induced ACF development and pathological changes in the colonic mucosal tissues, increased bone marrow MN cells and oxidative stress (glutathione depletion, lipid peroxidation) in rat colonic cells. The concomitant treatment of AOM with PomPE, PapPE or SE significantly ameliorated the cytotoxic effects of AOM.

**Conclusions:**

The results of this study provide *in-vivo* evidence that PomPE, PapPE and SE reduced the AOM-induced colon cancer in rats, through their potent anti-oxidant activities.

## Background

Natural food products are rich in phytochemicals, a biologically active group of compounds, which are classified according to their chemical structure into flavonoids, phenolic acids, coumarins, and tannins [1]. The beneficial health effects of polyphenols and flavonoids are considered primarily due to their antioxidant and chelating activities against various oxidative stress insults and to reduce environmental heavy metals toxicity [2]. In the past few years, numerous research activities were directed to investigate the ability of different natural food products to protect human cells against cancer-inducing agents, with an ultimate goal to develop new food products with preventive property against cancer [3].

Recent epidemiological studies revealed that regular consumption of fruits and vegetables improve overall human health and wellbeing by preventing non-communicable diseases including several types of cancer [4]. Cancer is considered as a leading cause of death worldwide accounting for 7.6 million death in the year 2005, the rate of colorectal cancer (CRC) has increased dramatically as compared to other types of cancers. This increase was attributed to environmental insults that synergize with genetics for the epidemic of CRC worldwide [5]. The international experts from the recent World Cancer Research Fund Report, 2008, concluded that CRC is a global public health problem [6,7]. Azoxymethane (AOM) induces colon cancer in experimental animals; in a mechanism that is mediated by glutathione (GSH) depletion and impairing total antioxidant capacity in colonic cells of rats [8,9].

GSH is the major intracellular antioxidant and it undergoes oxidation to the disulfide form (GSSG) and oxidized form, which scavenges reactive oxygen species (ROS) [10]. In healthy cells and tissues, more than 90% of the total glutathione pool is in the reduced form (GSH), and less than 10% exists in the disulfide form (GSSG), and a reduced GSH/GSSG ratio is considered an indication of oxidative stress [11]. Oxidative stress is a condition under which GSH, antioxidant enzymes (glutathione peroxidase, superoxide dismutase, and catalase), and dietary antioxidants (vitamin C, selenium, β carotene and vitamin E) are not counterbalancing the reactive oxygen species (ROS), and subsequently induce cellular damage by carcinogenic pathogenesis [2].

AOM-mediated carcinogenesis process involves mutagenicity by initiating chromosomal damage and induction of micronuclei (MN) cells [12,13]. AOM induced morphological changes were associated with aberrant crypt foci (ACF) development and DNA damage in proliferated cells [14,15]. Fruits or vegetables skin, and seaweed are less utilized low value biomaterials in Oman. All these biomaterials contain health beneficial functional components. Although pomegranate peel-extract (PomPE), papaya peel-extract (PapPE), and seaweed-extract (SE) are good sources of antioxidants, very limited works have been reported for their health functional benefits. Accordingly, the present study was performed to investigate the effects of PomPE, PapE and SE against AOM-induced carcinogenesis and oxidative stress in rat colon. Results of this study could enrich the ongoing chemoprevention research activities against colon malignancies.

## Methods

### Preparation of natural food products extracts

Fresh pomegranate (*Punica granatum L.*) peel, papaya (*Carica papaya L*.) peel and red seaweed (*Melanothamnus somalensis*) were cut into small pieces and homogenized with distilled water (10 g/100 ml) in an electric blender. Homogenates were agitated on a magnetic stirrer for 3 h at medium speed in absence of the light (i.e. beakers were covered with aluminum foil). All homogenates were then centrifuged separately at 6000 rcf for 30 min at 4°C using Harrier 18/80 refrigerated centrifuge (SANYO, MSE, UK). The extracts were filtered by Whatman filter paper # 1 (150 mm) and were stored at -40°C until used. The formal identification of the plant material was conducted as follow: Khalid Abdullah Al-Hashmi, Department of Marine Science and Fisheries, Sultan Qaboos University, identified seaweed; Amani Al-Rawahi, Department of Food Science and Nutrition, Sultan Qaboos University, identified Pomegranate; and Mohammad Shafiur Rahman and Mostafa Waly, Food Science and Nutrition, Sultan Qaboos University, identified papaya.

### Determination of total phenol and flavonoid contents

Total phenol content of PomPE, PapPE and SE extracts was determined with the Folin-Ciocalteu reagent, where different volumes (based on polyphenol concentration: 50, 100, and 150 μl) of the three natural extracts were added to 2.50 ml of Folin-Ciocalteu phenol reagent and 750 μl of Na_2_CO_3_ (7.5%, w/v) [16]. Distilled water was then added to bring the total volume to 5 ml and the mixture was incubated at room temperature (22°C) for 2 hours. The absorbance of all samples was measured at 765 nm using a UV–visible spectrophotometer. A standard curve was developed considering Gallic Acid as standard. Results were expressed as mg of Gallic Acid (GA) Equivalent/g dry-solids.

Flavonoids content was determined according to the aluminum chloride colorimetric assay; distilled water (4 mL) was added to 1 mL of each extract. Then, 5% NaNO_2_ (0.3 mL) was added, followed by addition of 10% AlCl_3_ (0.3 mL). The mixtures were incubated at ambient temperature for 5 min, and then 2 mL of NaOH (1 N) was added. Immediately, mixture volume was made up to 10 mL with distilled water. The mixture was thoroughly vortexed and the absorbance of the pink color was determined at 510 nm. A standard curve was developed considering catechin as standard. The results were expressed as mg catechin (CE) equivalents/100 g dry-solids.

### Evaluation of free radical scavenging capacity by DPPH photometric assay

The capacity of the PomPE, PapPE or SE extracts to scavenge 1,1-diphenyl-2-picrylhydrazyl (DPPH) free radicals was measured by a spectrophotmetric method as described earlier [17]. Briefly 50 μl of each extract, at different concentrations (μg/ml) were mixed with 50 μl of a DPPH methanolic solution (0.04 mg/ml). Absorbance was measured at 517 nm after 30 min of incubation at room temperature. Controls contained all the reaction reagents except the extract or 2,6-di-tert-butyl-4-hydroxytoluene (BHT) as a positive control. The free radical scavenging capacity of different samples was expressed as % DPPH inhibition, a higher % free radical scavenging activity value indicates a higher antioxidant activity and it was calculated as follow:

$$\%\text{DPPH}\;\text{inhibition}=\left[\left(\text{Absorbance}\;\text{of}\;\text{control}–\text{Absorbance}\;\text{of}\;\text{sample}\right)/\text{Absorbance}\;\text{control}\right]\times 100$$

### Evaluation of the antioxidant activity by the ABTS antioxidant assay

A colorimetric method using ABTS Antioxidant Assay Kit (Zenbio, Cat#AOX-1, USA) was used. The assay was based on the incubation of different extracts at different concentrations (5–300 μg/ml) with 2, 2′-azino-di-[3-ethylbenzthiazoline sulphonate] (6) diaammonium salt (ABTS). Peroxidase (methmyoglobin) and hydrogen peroxide produce the radical cation ABTS^+^ which was relatively stable blue-green color and absorbance was measured at 405 nm. Antioxidants present in the assayed extracts inhibited the oxidation of ABTS to ABTS^+^ (cause suppression of the color production), which was proportional to their concentration. The total antioxidant capacity of the assayed extracts was compared with Trolox standard, vitamin E analogue.

### Animals

Male Sprague–Dawley rats, aged 4 weeks were used in this experiment. The rats were housed in individual polypropylene cages and were provided with standard laboratory chow diet (Oman Mills, Muscat, Oman) and normal tap water *ad-libitum*. Rats were kept under standard conditions (i.e. temperature 22 ± 2°C, relative humidity 60%) and a 12 hr light:dark cycle. The protocol used in thus study was approved by the Animal Ethics committee at the Sultan Qaboos University (SQU/AEC/2010-11/1), and was conducted in accordance with international laws and policies [18].

### Experimental procedure

Eighty weanling male Sprague–Dawley rats (aged 4 weeks, with an average body weight of 50 ± 5 g) were randomly divided into 8 groups (10 rats/group). Control group was fed a basal diet; AOM-treated group was fed a basal diet and received AOM intra peritoneal injections, whereas the other six groups were fed PomPE, PapPE or SE via oral route (1.5 ml/day) in the presence or absence of AOM injection. The extract dose was 1.05 g fresh biomaterial per week (i.e. 21 g per kg body weight). PomPE, PapPE and SE extracts contained polyphenols 660, 266 and 33 mg GAE/kg BW for a week respectively, while these extracts contained flavonoids 142, 399 and 494 mg CEE/kg BW, respectively. Effective doses were selected similar to earlier works by our research groups (9, 10). All animals were continuously fed *ad-libitum*, body weight and food intake was recorded weekly for the whole duration of the experiment (16 weeks).

After 2 weeks from the last AOM injection, the animals were sacrificed by decapitation under diethyl ether anesthesia after an overnight fast and the colon tissues were removed for subsequent analysis: 1) Microscopic examination for aberrant crypt foci (ACF) numeration or any other morphological changes; 2) Biochemical analysis of the colonic tissue homogenates included GSH measurement, cellular peroxide production and myeloperoxidase activity; 3) Mutagenicity evaluation by examining bone marrow cells with micronuclei (MN).

### AOM injections

At week six, the rats in the control group were given 1 ml intra peritoneal injection of 0.9% physiological saline once a week for 2 weeks and the rats in the AOM-injected group were given 2 intra peritoneal injections of AOM (Sigma Chemical Co., St. Louis, MI) dissolved in physiological saline once a week (15 mg/kg body weight) for 2 weeks, this dose was based on previous studies from our research group.

### Bone marrow micronuclei assay

Immediately after the animals were sacrificed, femur and tibia were dissected and freed from adherent tissues. The bone marrow was flushed out by injecting filtered fetal calf serum (FCS) using a syringe. The collected cells were centrifuged at 390 g for 5 min, supernatant was discarded and the cells were re-suspended in the FCS. A small drop of the re-suspended cell suspension was spread on a glass slide, fixed in absolute methanol for 1–3 min and air-dried at room temperature for 5–10 min. The slides were stained for 15 min in phosphate buffered saline (0.1 M; pH 6.8) containing 10 μg/ml of acridine orange (freshly prepared), rinsed in the same buffer for 15 min and allowed to dry in the dark at room temperature. The slides were scored immediately under 10007× magnification using a fluorescence microscope (Olympus BX51) [19].

The cytotoxicity index (CI) was determined as the ratio of polychromatic erythrocytes (PCE) to normochromatic erythrocytes (NCE) (PCE/NCE), smears were stained with Giemsa (Merck) (0.08% in phosphate buffer) and one thousand polychromatic erythrocytes (PCE) were examined from each animal [20].

### Colon preparation

The colons were carefully removed from rats and were kept on a glass plate in ice jackets. The colons were then opened longitudinally, rinsed with ice-cold physiological saline, and were sectioned longitudinally into two halves of equal width and were spread out with flat mucosal side up. The mucosal layer from one half was removed by scraping and immediately homogenized. The other half was fixed flat in 10% buffered formalin (Fisher Scientific, Fair Lawn, NJ) between two filter papers for one week before Aberrant Cypt Foci (ACF) enumeration.

### Aberrant Cypt Foci (ACF) enumeration and microscopic examination

ACF was commonly accepted precursor lesions for colonic tumors and the method used for ACF enumeration was followed as described in previous studies [8,9]. Fixed colons were stained with 0.2% methylene blue in Kreb’s ringer bicarbonate buffer for 20 minutes in a Petri dish and rinsed with physiological saline. After staining, the colons were placed (i.e. mucosal surfaces) up on a slide, examined with a light microscope under ×40 magnification and scored for ACF. In brief, the ACF were distinguished from normal crypts by their darker stain, enlarged and slightly elongated size, thick epithelial lining, slightly elongated cryptal opening and their slit shapes. The total number of ACF was recorded for all examined colons. The rest of the sample was fixed in 10% formalin overnight. Following tissue processing and paraffin embedding, sections were cut at 3 μm thickness and placed on glass slides; these were then stained with haematoxylin and eosin for colon architecture histology under light microscope examination (magnification ×400).

### Scraped colonic mucosa homogenization

The colon mucosal layer scrapings of each rat (~50 mg) were immediately homogenized in 1 mL of 100 mM potassium phosphate buffer (pH 7.2) by a glass-Teflon homogenizer with an ice-cold jacket and centrifuged at 100,000 g at 4°C for 60 minutes. The resulting supernatant was used for the determination of protein content, and performing biochemical analyses.

### Analysis of protein content

Protein content of colon tissues was assayed by the method of Lowry *et al*. [21], using bovine serum albumin as standard and protein content was expressed as mg/ml of sample.

### Evaluation of lipid Peroxidation

In this study, lipid peroxidation in the colon tissue homogenates was determined by measuring the production of malondialdehyde (MDA) using a commercial kit, (Thiobarbituric Acid Reactive Substances (TBARS) Assay Kit from Cayman Chemical Item Number 10009055). The TBARS Assay was conducted based on the manufacture’s instruction, and in brief MDA reacts with thiobarbituric acid (TBA) forming a colored product measured at absorbance 532 nm.

### Dichlorofluorescein fluorescence assay (DCF)

The dichlorofluoresceine fluorescence assay was used to measure cellular peroxide production and other reactive species [22]. Aliquots of colon samples were added to a medium containing Tris–HCl buffer (0.01 mM, pH 7.4) and dichlorofluoresceine diacetate (7 μM). The medium was incubated in dark for 1 h until the fluorescence measurement (excitation at 488 nm and emission at 525 nm, with both slit widths at 1.5 nm). Oxidized dichlorofluoresceine was determined using a standard curve of oxidized dichlorofluoresceine and results were expressed as μmol of oxidized DCF/mg protein [23].

### Glutathione (GSH/GSSG) assay

The supernatant was separated into two aliquots for measurement of GSH and GSSG, 100 μL was transferred to fresh eppendorf tubes and 2 μL of monochlorobimane (25 mmol/L) and 2 μL of glutathione-S-transferase reagent were added, as provided by a commercial kit (Biovision, Mountain View, CA, USA, Catalog # K251). After 30 minutes of incubation at 37°C, the samples and standards were read in a fluorescence plate reader at 380/460 nm. GSH and GSSG content was determined by comparison with values from a standard curve using freshly prepared GSH and GSSG, which was normalized with protein content in the colonic mucosa tissue homogenates. Results were expressed as ratio of GSH/GSSG.

### Myeloperoxidase activity assay

Myeloperoxidase activity was determined in colon supernatant fraction, briefly; a sample of the colon was added to a medium containing potassium phosphate buffer (50 mM, pH 6.0) containing hexadecyltrimethylammonium bromide (0.5%) and N, N, N0, N0-tetramethylbenzidine (1.5 mM). The kinetic analysis of myeloperoxidase was started after the addition of hydrogen peroxide (0.01%), and the color reaction was measured at 655 nm at 37°C [24].

### Statistical analysis

Statistical analysis was performed using GraphPad Prism (version 5.03; GraphPad Software Inc. San Diego, CA). The results were expressed as means ± standard deviation (SD). The statistical analysis was performed using one way analysis of variance (ANOVA) followed by Tukey’s test, Student’s unpaired *t*-test for means comparisons, and a P-value of less than 0.05 is considered significant.

## Results

### Antioxidant properties of the extracts

**Figure 1:**
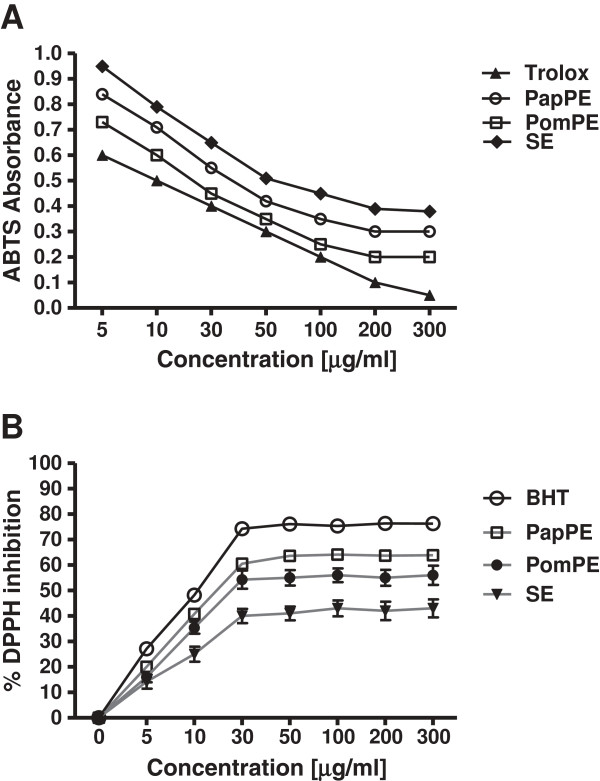
**Radical scavenging activity of the extracts as evaluated by the DPPH and ABTS assays. (A)** BHT, PapE, PomPE and SE scavenged DPPH free radicals formation in a dose-dependent manner, and reached plateau at a concentration of 30 μg/ml. The dose-dependent effect of PapPE was higher than PomPE and SE. Results are the means ± SD of 6 measurements. **(B)**: Free radicals scavenging ability of vitamin E analog (Trolox), PapPE, PomPE and SE against ABTS radical formation. PapPE, PomPE and SE showed inhibition of ABTS radical formation in a dose-dependent manner. The IC_50_ of PapPE was comparable for Trolox standard, and significantly higher than PomPE and SE, *P* < 0.05. Results are the means ± SEM of 6 measurements.

The moisture contents of the papaya skin, pomegranate skin and seaweed contained 72.0, 76.0, and 86.5 g/100 g sample (wet basis), respectively. The phytochemical analysis of the biomaterials showed the presence of various quantities of polyphenols and flavonoids, and it was observed that PapPE showed the highest contents of polyphenols, SE contained the highest flavonoids as compared to others (Table 1). The radical scavenging activity of the extracts was evaluated by the DPPH and ABTS assays. PapPE was a potent radical scavenger as compared to PomPE and SE, with a decrease in the DPPH percentage and ABTS radicals’ formations (Figure 1A & B). Polyunsaturated fatty acids (PUFA) are essential components of cell membrane phospholipids and excessive ROS may cause oxidative damage of PUFA leading to cell death. The occurrence of malondialdehyde (MDA), a secondary end product of the oxidation of polyunsaturated fatty acids, was considered a useful index of general lipid peroxidation.

**Table 1:** Quantitative assessment of total polyphenols and flavonoids content of the biomaterials

**Group** **Water content or bioactive component**	**PapPE**	**PomPE**	**SE**
Water content	77.4 ± 0.9*	73.6 ± 1.1*	86.5 ± 0.8*
(g/100 g fresh sample)			
Total polyphenols	139 ± 2*	56 ± 6*	7 ± 2*
(mg GAE/g dry-solids)			
Flavonoids	30 ± 1*	84 ± 1*	104 ± 3*
(mg CE/g dry-solids)			

Values were expressed in means ± SD. PapPE has the highest the total polyphenols and SE has highest flavonoids content as compared to others, *symbol indicates significant difference in row at P<0.05.

**Figure 2:**
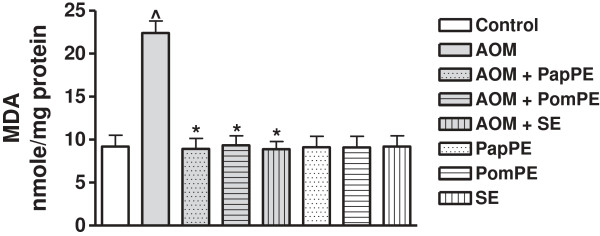
**Lipid peroxidation as determined by MDA level in colonic tissue homogenates of rats exposed to AOM and in the presence or absence of PapPE, PomPE and SE.** ^Significantly higher than control group. *Significantly lower than AOM group, *P* <0.05. PapPE, PomPE and SE were similar to control group with no statistical differences.

Figure 2 shows the effect of AOM on the induction of lipid peroxidation in colonic cells as determined by MDA level (nmole/mg protein). It was observed that, MDA levels in colon tissue homogenates of rats treated with AOM was significantly increased (22.4 ± 1.4) as compared to control group (9.2 ± 1.3), *t* = 6.91, *P* < 0.0001. However the combined treatment of AOM with PapPE, PomPE and SE induced a significant decrease in MDA level to reach a comparable level to the control group (8.9 ± 1.2, 9.3 ± 1.1, and 8.9 ± 0.9, respectively), F = 32.90, R square = 0.8315, *P* < 0.0001. There was no significant difference between control, PapPE, PomPE and SE groups, *P* >0.05.

### Dichlorofluorescein fluorescence assay (DCF)

**Figure 3:**
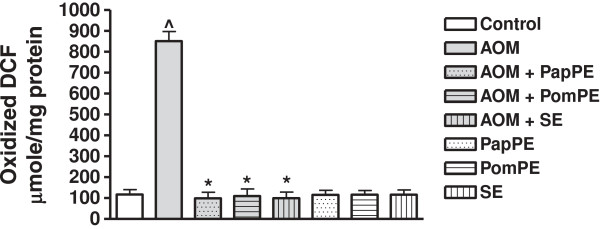
**Peroxide protein formation as determined by Oxidized DCF level in colonic tissue homogenates of rats exposed to AOM and in the presence or absence of PapPE, PomPE and SE.** ^Significantly higher than control group. *Significantly lower than AOM group, *P* <0.05. PapPE, PomPE and SE were similar to control group with no statistical differences.

The dichlorofluoresceine fluorescence (DCF) assay was used to measure cellular peroxide production, and oxidized DCF-protein. The AOM treatment showed a significant increase in oxidized DCF levels as compared to controls group. Meanwhile the extracts prevented the formation of oxidized DCF proteins (Figure 3).

### Micronuclei (MN) cells counting and cytotoxicity index

**Figure 4:**
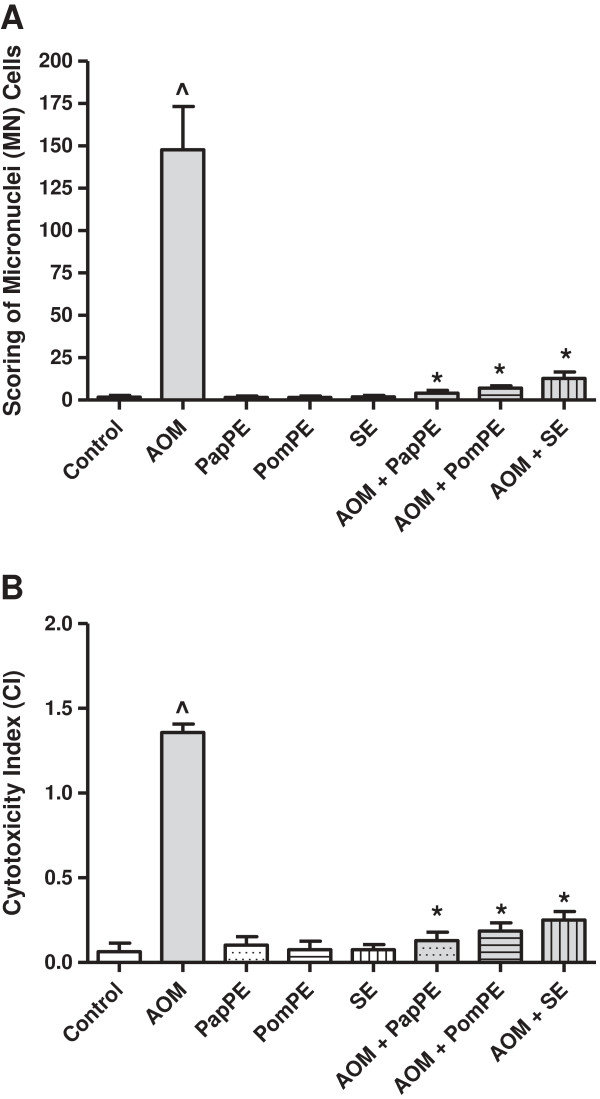
**Micronuclei cells counting and cytotoxicity index. (A)** Scoring of micronuclei (MN) cells, indicator of genotoxicity, among control, AOM and treated groups. ^Significantly higher as compared to control group. *Significantly lower than AOM-injected group, *P* < 0.05. **(B)** The cytotoxicity index (CI) was determined as the ratio of polychromatic erythrocytes (PCE) to normochromatic erythrocytes (NCE), ^Significantly higher as compared to control group. *Significantly lower than AOM-injected group, *P* < 0.05.

Micronucleus assay was used as a prognostic test for detection of chromosomal alterations/damage relevant to carcinogenesis. The rodent bone marrow MN test was most widely used as an *in-vivo* assay for identification of genotoxic effects of different carcinogenic agents [19,20]. The cytotoxicity index (CI) was determined as the ratio of polychromatic erythrocytes (PCE) to normochromatic erythrocytes (NCE). Results are presented on Figures 4A & B.

### GSH/GSSG ratio

**Figure 5:**
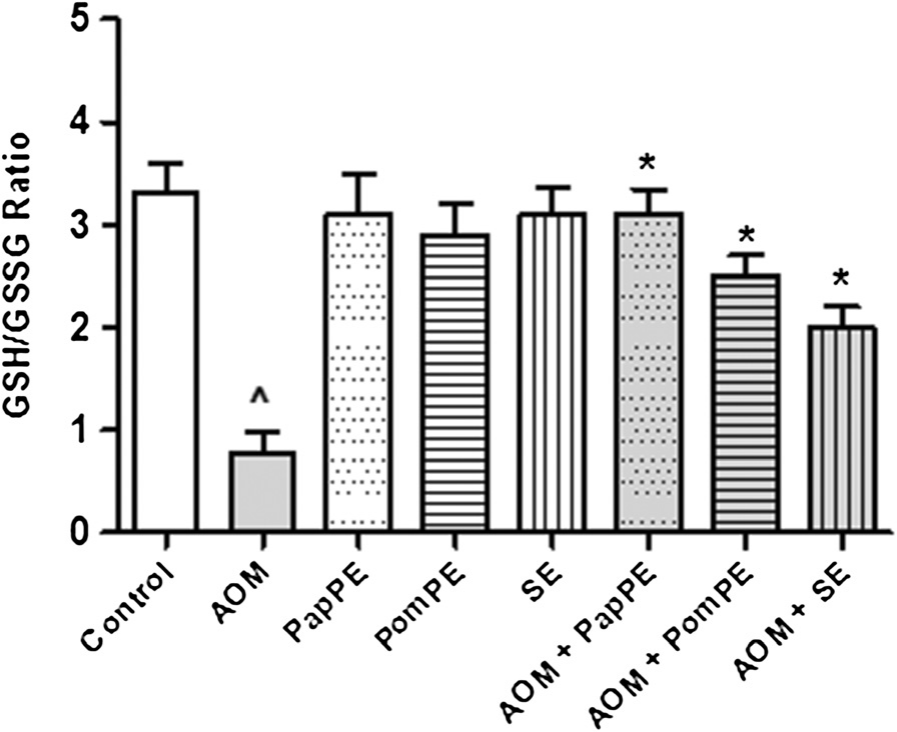
**The oxidative stress index was determined as the ratio of GSH/GSSG.** ^Significantly higher as compared to control group. *Significantly lower than AOM-injected group, *P* < 0.05.

Figure 5 represents that the AOM group showed significant changes on oxidative markers, which was indicated by a decrease on GSH/GSSG ratio in relation to respective control animals. However, extracts protected treated animals against the damage induced by AOM. It is worth to mention that PapPE was the most potent extract that significantly decreases the AOM-mediated impairment of GSH/GSSG ratio.

### Myeloperoxidase activity assay

**Figure 6:**
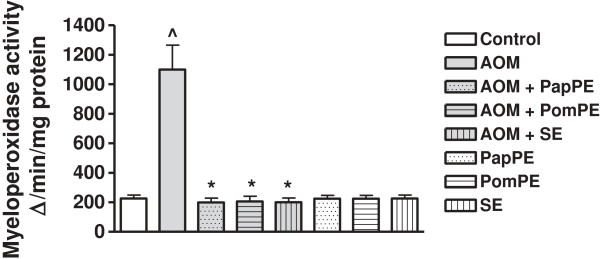
**The inflammatory response was measured by myeloperoxidase activity.** ^Significantly higher as compared to control group. *Significantly lower than AOM-injected group, *P* < 0.05. PapPE, PomPE and SE were similar to control group with no statistical differences

Myeloperoxidase is pro-inflammatory mediator, and its activity was increased during inflammation. The animals that were treated with AOM showed significant changes of inflammatory response marker (i.e. increase of myeloperoxidase activity) as compared to control animals. The extracts significantly decreased the AOM-mediated inflammation. The PapPE showed highest protective effects as compared to PomPE and SE (Figure 6).

### Microscopic examination

**Figure 7:**
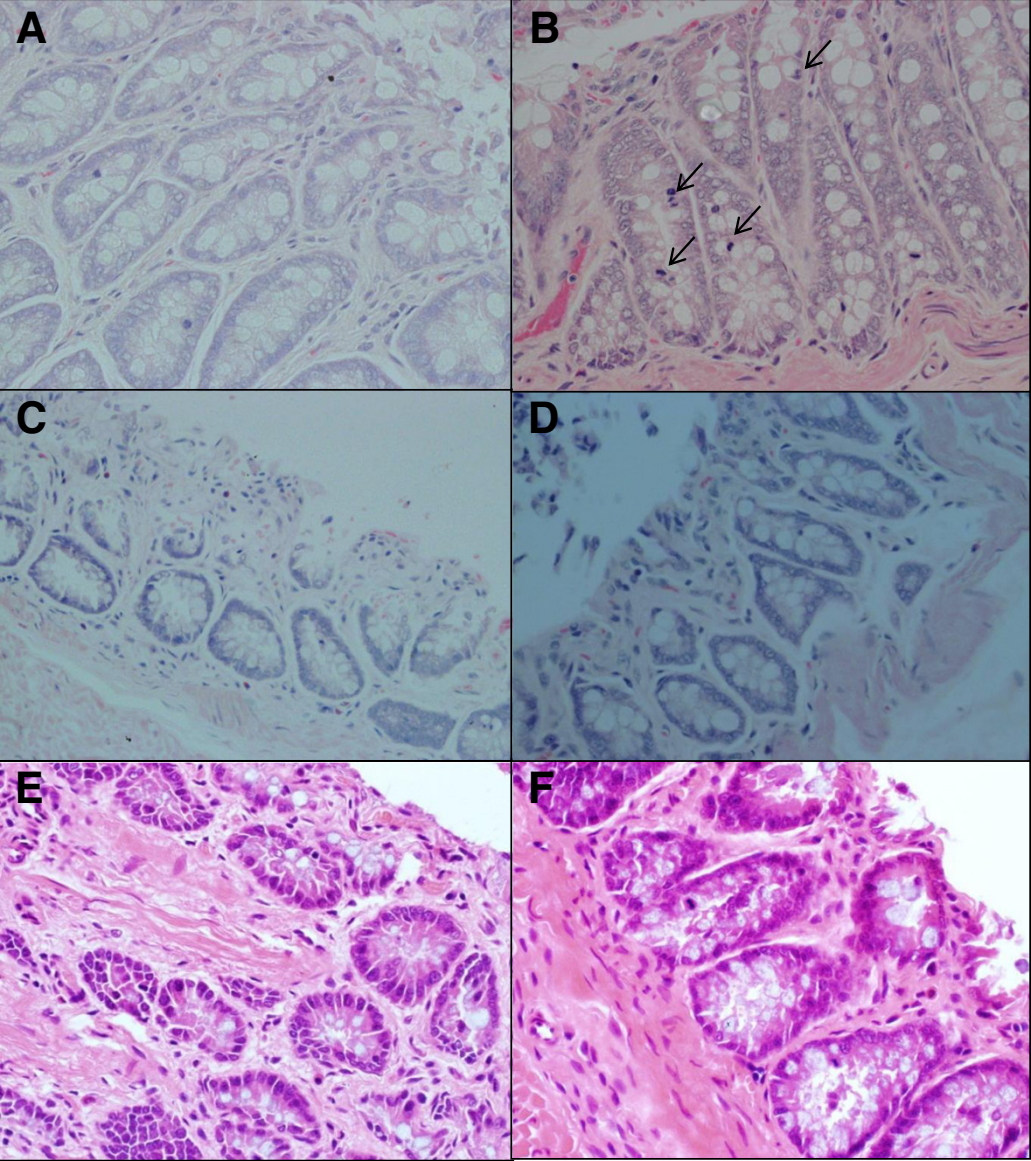
**Representative section of colon architecture histology under the light microscope after haematoxylin and eosin staining. (A)** Control rats with normal colon architecture. **(B)** azoxymethane-treated rats showed colonic mucosa with preserved architecture and lined by unremarkable epithelium in nearly two thirds of the examined area. There is an increase in mitotic activity in the crypts (arrows) compared to the control rats which may suggest regeneration in response to AOM-induced-injury. **(C)**, **(D) & (E)** azoxymethane-treated rats in the presence of extracts (PapPE, PomPE and SE respectively) showed dramatic improvement in the histologic appearance similar to the control rats, There was surface autolysis in these samples but deeper crypts appear unremarkable. **(F)** A representative section for PapPE, PomPE or SE treated groups; showed histological appearance similar to the control rats.

The size of the examined ACF was medium, 4–6 aberrant crypt/foci, and as illustrated in Table 2; PapPE, PomPE and SE-administration inhibited the AOM-induced ACF development as compared to the control group. Meanwhile; PapPE, PomPE and SE were similar to control group with no statistical differences (data is not presented on the table). The examination of haematoxylin and eosin stained sections was reported in Figure 7; for all samples the architecture of the mucosa was generally preserved except for AOM-injected rats which showed increased mitotic activity in the crypts when compared with the samples taken from normal controls and with those treated with extracts (PapPE, PomE and SE) in the presence of AOM.

**Table 2:** Aberrant crypt foci (ACF) enumeration and distribution

**Groups**	**ACF count in proximal colon**	**ACF count in distal colon**
Control	0.00 ± 0.00	0.00 ± 0.00
AOM	6.89 ± 2.61^#^	39.64 ± 11.23^#^
AOM + PapPE	0.52 ± 0.12^*^	3.54 ± 0.09^*^
AOM + PomPE	2.34 ± 0.13^*^	8.64 ± 2.11^*^
AOM + SE	4.51 ± 0.79^*^	11.76 ± 1.93^*^

Values were expressed in means ± SD. ^#^Significantly higher than control group. *Significantly lower than AOM group, *P* < 0.05.

## Discussion

The concept of cancer prevention using diet containing naturally occurring is gaining increased attention. In this line, different types of fruits and vegetables have been re-evaluated and recognized as valuable sources of phytochemicals (polyphenols and flavonoids compounds) and labeled as bioactive components [25]. The application of such bioactive plant components may have potential in preventing a range of chronic disorders, including cancer [26].

In our study, the observed anti-carcinogenic properties of the PapPE, PomPE and SE, may be attributed to their antioxidant phytochemicals constituents, these extracts acted as a colon protective agent against the damage caused by AOM-induced insult, and this observation was in consistent with previous studied reported by our group [8,9]. The antioxidant potential properties of the extracts prevented the generation of ROS in the colinic cells as presented by its ability to quench and scavenge DPPH and ABTs radicals’ formation (Figure 1A & B). We postulated that the presence of extracts with AOM by pre and post treatment normalized the levels of the colonic intracellular antioxidant capacity to nearly normal. The observed chemoprevention effect of the assayed extracts was exerted by its capacity as a potent antioxidant agents, hence boosting the colonic cellular antioxidant capacity of rats treated with PapPE/AOM, PomPE/AOM and SE/AOM versus rats treated with AOM.

Another significant mechanism displayed by PapPE, POmPE and SE was the protection of plasma membrane of colonic cells against AOM-mediated cellular damage, this protective mechanism of the extracts was identified by measuring the MDA (degradation end product of oxidation of cell membrane phospholipids) and DCF (cellular protein peroxide production), (Figures 2 and 3). The increased levels of MDA and oxidized DCF indicated colonic cellular oxidative damage. The exposure of AOM induced a marked increase in MDA formation and DCF release in colonic tissues, but administration of the extracts (PapPE, PomPE and SE) significantly reduced MDA and DCF, near normal level. This was essential for colonic tissue integrity and healing process of the colonic mucosa against AOM-mediated carcinogenesis [27].

To evaluate the oxidative status, we looked for GSH which constitutes one of the major intracellular antioxidant index used to assess oxidative stress. The antioxidant effects of the extracts were evidenced by high GSH/GSSG ratio [the increased levels of reduced glutathione (GSH) associated with decreased levels of oxidized glutathione (GSSG)] (Figure 5). This data revealed that the phytochemicals present in the extracts could act as antioxidants to decrease the reactive oxygen species (ROS) levels, which were elevated due to the mucosal damage caused by AOM. Several investigators demonstrated that AOM induced ROS in colonic cells was primarily decreased the activity of antioxidant enzymes and depleted intracellular concentrations of GSH [8,9,14].

It is suggested that AOM, as an oxidative stress insult, initiates genotoxicity. Our study showed that AOM-treatment significantly increased the percentage of MN cells in bone marrow cells, indicating the chromosomal aberrations; while rats injected with AOM and received PapPE, PomPE or SE showed a significant reduction in MN cells and the cytotoxic index (Figures 4A & B). Thus, we suggested that AOM possesses a genotoxic effect and that the treatment with the extracts combat the AOM-mediated genotoxicity by inhibiting microsomal activation or by protecting DNA strands from the electrophilic metabolite of the mutagen, AOM. The extracts may inhibit several metabolic intermediates and reactive oxygen species (ROS) formed during the process of microsomal enzyme activation, which are capable of breaking DNA strands.

The marked inflammatory effect, high myeloperoxidase activity, associated with the AOM injection suggested that the AOM-induced oxidative stress was partly mediated by an inflammatory response; this finding is in line of the notion that the inflammatory process, was a part of the cells natural defense against tissue damage, which was generally associated with oxidative stress [28,29]. Our study provided direct evidence that PapPE, PomPE and SE possessed a marked anti-inflammatory effect, as achieved by the decrease in the pro-inflammatory mediator, myeloperoxidase activity in the AOM-treated rats (Figure 6).

The increased proliferative activity seen in the examined colon samples for AOM-injured rats suggested a form of response to earlier or ongoing injury. The epithelial cells lining of the crypts showed an increased turn over, which most likely represented a regenerative phenomenon due to AOM-mediated mitogenic effect. On the other hand, AOM-injected rats received the extracts (PapPE, PomPE and SE) showed a less mitotic activity, which indicated a cytoprotective effect. There was no significant mitotic activity in control and PapPE, PomPE and SE treated groups (Figure 7).

## Conclusion

In conclusion, PapPE, PomPE and SE extracts exhibited anti-carcinogenic and anti-inflammatory effect against AOM-induced cytotoxicity in rat colon. PapPE was more potent in its protective effect as compared to PomPE or SE. The observed chemopreventive effect of these natural extracts on AOM-mediated carcinogenesis and oxidative stress in rat colon was attributed to its bioactive components (polyphenols and flavonoids).

## Competing interests

The authors declare no competing interest for this study.

## Authors’ contributions

MW and SR conceptualized the study protocol and supervise the whole project. All authors have made equal contribution to the manuscript writing and data interpretation. All authors read and approved the final manuscript.

## Pre-publication history

The pre-publication history for this paper can be accessed here:

http://www.biomedcentral.com/1472-6882/14/60/prepub
